# Respiratory symptoms, preventive practices, and antibiotic use during Hajj: A post-pandemic cross-sectional survey among Malaysian pilgrims

**DOI:** 10.1016/j.ijregi.2026.100945

**Published:** 2026-07-10

**Authors:** Nurul Azmawati Mohamed, Shalinawati Ramli, Nuurain Amirah Mohd Razi

**Affiliations:** Faculty of Medicine and Health Sciences, Universiti Sains Islam Malaysia, Nilai, Malaysia

**Keywords:** Hajj, Mass gathering, Respiratory symptoms, Antibiotic use, Malaysia, Preventive practices

## Abstract

**Objectives:**

Hajj is a major international mass gathering associated with high respiratory symptom burden. After coronavirus disease 2019, preventive behaviors such as vaccination, mask use, hand hygiene, gargling, and cough etiquette may have changed. This study described respiratory symptoms, preventive practices, antibiotic use, and functional impact among Malaysian Hajj pilgrims in 2023.

**Methods:**

A cross-sectional online survey was conducted from September to December 2023 among Malaysian adults who performed Hajj in 2023. Data included comorbidities, vaccination status, preventive practices, respiratory symptoms, antibiotic use, and impact on daily activities. Symptoms were self-reported and not clinically or microbiologically confirmed. Descriptive and logistic regression analyses were performed.

**Results:**

Among 743 respondents, 619 (83.3%) reported at least one respiratory symptom. Common symptoms were cough with phlegm (70.0%), runny nose (62.0%), sore throat (57.7%), and fever (43.5%). Most respondents received both influenza and pneumococcal vaccines (77.8%). Mask use (79.5%), cough etiquette (67.8%), and handwashing (64.3%) were common, whereas regular hand sanitizer use (25.6%) and gargling were less frequent. Antibiotics were used by 58.8% of symptomatic respondents. Chronic disease increased symptom odds, whereas frequent hand sanitizer use reduced odds.

**Conclusion:**

Respiratory symptoms remained common post-pandemic despite high vaccination uptake and preventive practices. Findings support strengthened pre-Hajj health optimization, respiratory hygiene education, and prudent antibiotic use.

## Highlights

• Post-pandemic Hajj showed high respiratory symptom burden.
• Chronic disease was linked to higher respiratory symptom reporting.
• Hand sanitizer use was linked to lower respiratory symptom reporting.
• Antibiotic use was common despite unconfirmed respiratory infection.
• Respiratory symptoms disrupted worship activities during Hajj.

## Introduction

Hajj is one of the largest annual religious mass gatherings in the world and represents a unique intersection among faith, health, mobility, and public health preparedness. Each year, Muslim pilgrims from diverse geographical, cultural, and health backgrounds gather in Saudi Arabia to perform a sequence of physically and spiritually demanding rituals. During the 2023 Hajj season, nearly two million pilgrims participated, marking the resumption of large-scale international pilgrimage after the coronavirus disease 2019 (COVID-19) pandemic.

Respiratory symptoms are among the most frequently reported health problems during Hajj. Crowding, prolonged close contact, shared accommodation, transport congestion, communal dining, and exposure to pilgrims from multiple countries increase the likelihood of respiratory symptom transmission. A survey among French pilgrims found that more than 80% experienced respiratory symptoms and almost half used antibiotics during pilgrimage [1]. Earlier Malaysian data also reported a high occurrence of respiratory illness among pilgrims [2]. Given that most pilgrims are elderly, they are more prone to get severe respiratory tract infections (RTIs) and require hospitalization. Data among Indian pilgrims showed that more than half of hospital admissions were caused by overwhelming RTIs [3,4]. Although these studies demonstrate that respiratory symptoms are common during Hajj, post-pandemic data from Malaysian pilgrims remain useful because health behaviors, vaccine uptake, mask use, and risk perception may have changed after COVID-19.

In addition to health service use and antibiotic prescribing, respiratory symptoms may affect pilgrims’ ability to complete daily and pilgrimage-related activities. Understanding symptom burden, preventive practices, antibiotic use, and associated factors can inform pre-Hajj counseling, targeted prevention for higher-risk pilgrims, and mass gathering health preparedness. This study aimed to describe self-reported respiratory symptoms, preventive practices, antibiotic use, and functional impact among Malaysian Hajj pilgrims in 2023 and to explore factors associated with reported symptoms.

## Methods

This cross-sectional online survey was conducted from 1 September 2023 to 31 December 2023, among Malaysian adults who performed Hajj in 2023. The sample size was calculated using Epi Info based on an estimated population of 30,000 Malaysian Hajj pilgrims, a 95% confidence level, a 5% margin of error, and a 50% response distribution. After allowing for a 20% non-response rate, the minimum required sample size was 400. Eligible respondents were Malaysian citizens aged 18 years and older who had performed Hajj in 2023.

### *Study instrument*

Data were collected using a self-administered Google Forms questionnaire. The survey link was distributed through WhatsApp groups created for Malaysian Hajj pilgrims in 2023. The first page of the form contained study information and a consent statement. Respondents could proceed only after indicating their agreement to participate. Participation was voluntary, anonymous, and without compensation. The questionnaire consisted of two sections: sociodemographic and Hajj-related information and self-reported respiratory symptoms, antibiotic use, preventive practices, and perceived impact on daily and worship-related activities.

For this study, self-reported respiratory symptoms were defined as the presence of at least one of the following symptoms during the Hajj period: cough with phlegm, cough without phlegm, runny nose, sore throat, fever, blocked nose, hoarseness of voice, difficulty breathing, or chest pain during inspiration. These symptoms were based on respondent recall and were not confirmed by clinical examination, medical records, or microbiological testing. Therefore, the outcome is reported as self-reported respiratory symptoms rather than clinically confirmed RTI.

### *Quality control*

The questionnaire was reviewed by subject-matter experts in public health, microbiology, infectious diseases, and epidemiology to assess item relevance, clarity, and appropriateness. Face validity was assessed through pretesting among 30 respondents with characteristics broadly similar to the target population. Feedback was obtained on clarity, wording, comprehension, completion time, and cultural suitability. Items were revised accordingly before final distribution. Reliability testing showed an intra-class correlation coefficient of 0.822 for selected respiratory symptom-related items, indicating good agreement.

### *Statistical analysis*

Data were analyzed using IBM SPSS Statistics version 26. Descriptive statistics were used to summarize sociodemographic characteristics, comorbidities, preventive practices, self-reported respiratory symptoms, antibiotic use, and impact on daily and worship-related activities. Categorical variables were presented as frequencies and percentages, whereas continuous variables were presented as mean and SD. The primary outcome was the presence of at least one self-reported respiratory symptom during Hajj. Associations between categorical variables and self-reported respiratory symptoms were assessed using the chi-square test or Fisher’s exact test, where appropriate. Continuous variables such as age and body mass index were compared using the independent samples *t*-test. Variables with *P* < 0.25 in bivariate analysis, together with clinically relevant variables, were considered for inclusion in the multivariable logistic regression model. Logistic regression results were reported as adjusted odds ratios (AORs), 95% confidence intervals (CIs), standard errors, test statistics, and *P*-values. *P* < 0.05 was considered statistically significant.

## Results

### *Sociodemographic characteristics*

A total of 743 Malaysian Hajj pilgrims participated in this survey. The mean age of respondents was 51.3 years (SD 9.9; range 18-96 years). More than two-thirds of respondents were female, 510 of 743 (68.6%), whereas 233 of 743 (31.4%) were male.

### *Comorbidities*

The most frequently reported comorbidities were hypertension, reported by 183 respondents (24.6%), followed by hypercholesterolemia, 122 (16.4%), and diabetes mellitus, 93 (12.5%). Overall, 217 respondents (29.2%) reported having at least one chronic disease.

### *Preventive measures*

Preventive practices varied among respondents. Most respondents reported receiving at least one vaccine other than the mandatory meningococcal vaccine, 687 of 743 (92.5%). Of these, 578 respondents (77.8%) reported receiving both influenza and pneumococcal vaccines. Other commonly reported preventive practices included wearing a face mask in public places, 591 (79.5%); taking supplements, 566 (76.2%); practicing cough etiquette, 504 (67.8%); and washing hands with water and soap, 478 (64.3%). However, regular use of hand sanitizer was reported by only 190 respondents (25.6%). Gargling practices were also uncommon, with only 108 respondents (14.5%) reporting gargling with water and 170 (22.9%) reporting gargling with antiseptic mouth rinse. The distribution of comorbidities and preventive practices is presented in Table 1.

**Table 1:** Comorbidities and preventive practices among Malaysian Hajj pilgrims.

Variables	No n (%)	Yes n (%)
Comorbidities		
Diabetes mellitus	650 (87.5)	93 (12.5)
Hypertension	560 (75.4)	183 (24.6)
Hypercholesterolemia	621 (83.6)	122 (16.4)
Heart disease	727 (97.8)	16 (2.2)
Chronic renal disease	740 (99.6)	3 (0.4)
Chronic lung disease	743 (100)	0
Cancer	738 (99.3)	5 (0.7)
Preventive measures		
Vaccinated other than meningococcal	56 (7.5)	687 (92.5)
Influenza only	637 (93.3)	50 (6.7)
Pneumococcal only	628 (92.1)	59 (7.9)
Influenza and pneumococcal	109 (22.2)	578 (77.8)
Supplement use	177 (23.8)	566 (76.2)
Hand hygiene practice (wash hands with water and soap)	265 (35.7)	478 (64.3)
Frequent hand sanitizer use	553 (74.4)	190 (25.6)
Face mask use in public places	152 (20.5)	591 (79.5)
Cough etiquette	239 (32.2)	504 (67.8)
Gargle with water	635 (85.5)	108 (14.5)
Gargle with antiseptic mouth rinses	573 (77.1)	170 (22.9)
Eat balanced diet	459 (61.8)	284 (38.2)

### *Self-reported respiratory symptoms during Hajj*

Overall, 619 of 743 respondents reported at least one respiratory symptom during Hajj, giving a proportion of 83.3% among survey respondents. Given that symptoms were based on retrospective self-report and were not clinically or microbiologically confirmed, these findings are reported as self-reported respiratory symptoms rather than confirmed RTI. Among the 619 symptomatic respondents, 276 (44.6%) reported one episode of respiratory symptoms, whereas 203 (32.8%) reported two episodes. A smaller proportion reported more frequent episodes, including 73 (11.8%) who reported three episodes, 20 (3.2%) who reported four episodes, 4 (0.7%) who reported five episodes, and 43 (6.9%) who reported six episodes. Sixteen respondents required hospitalization for severe respiratory symptoms. This represented 2.2% of all respondents and 2.6% of respondents who reported respiratory symptoms. The frequency of self-reported respiratory symptom episodes and antibiotic use among symptomatic respondents is presented in Table 2.

**Table 2:** Frequency of self-reported respiratory symptom episodes and antibiotic use among symptomatic respondents.

Number of symptom episodes	Frequency of symptom episodes, n (%)	Frequency of antibiotic use for respiratory symptoms					
		0, n (%)	1, n (%)	2, n (%)	3, n (%)	4, n (%)	5, n (%)
1	276 (44.6)	156 (56.5)	101 (36.6)	16 (5.8)	3 (1.1)	0 (0)	0 (0)
2	203 (32.8)	56 (27.6)	83 (40.9)	58 (28.6)	5 (2.5)	1 (0.5)	0 (0)
3	73 (11.8)	19 (26)	31 (42.5)	14 (19.2)	7 (9.6)	1 (1.4)	1 (1.4)
4	20 (3.2)	10 (50)	7 (35)	2 (10)	1 (5)	0 (0)	0 (0)
5	4 (0.7)	0 (0)	1 (25)	2 (50)	0 (0)	0 (0)	1 (25)
6	43 (6.9)	14 (32.6)	16 (37.2)	5 (11.6)	6 (14)	0 (0)	2 (4.7)
Total	619 (100)	255 (41.2)	239 (38.6)	97 (15.7)	22 (3.6)	2 (0.3)	4 (0.6)

### *Antibiotic use among symptomatic respondents*

Among the 619 respondents who reported respiratory symptoms, 255 (41.2%) did not receive antibiotics. Antibiotic use was reported once by 239 respondents (38.6%), twice by 97 respondents (15.7%), three times by 22 respondents (3.6%), four times by two respondents (0.3%), and five times by four respondents (0.6%). Among those who reported only one episode of respiratory symptoms, more than half, 156 of 276 (56.5%), did not receive antibiotics. In contrast, antibiotic use became more frequent among respondents who reported repeated respiratory symptom episodes. For example, among those who reported three episodes, 54 of 73 (74.0%) received antibiotics at least once.

### *Types of respiratory symptoms and impact on daily activities*

Among the 619 symptomatic respondents, the most common symptoms were cough with phlegm, reported by 433 respondents (70.0%), followed by runny nose, 384 (62.0%); sore throat, 357 (57.7%); fever, 269 (43.5%); blocked nose, 258 (41.7%); and hoarseness of voice, 244 (39.4%). Less commonly reported symptoms included difficulty breathing, 44 (7.1%), and chest pain during inspiration, 43 (6.9%). Respiratory symptoms also affected worship-related activities. Persistent cough affected prayer among 285 respondents (46.0%) and Quran recitation among 213 respondents (34.4%). A total of 203 respondents (32.8%) reported that their symptoms made it challenging to participate in spiritual practices. Other reported impacts included urinary incontinence during coughing, 152 (24.6%); loss of appetite, 134 (21.6%); and lack of energy, 133 (21.5%). However, 122 respondents (19.7%) reported no impact on daily activities. The distribution of respiratory symptoms and their impact on daily worship-related activities is presented in Table 3.

**Table 3:** Self-reported respiratory symptoms and implications on daily activities among symptomatic respondents.

Self-reported respiratory symptoms and implications	n (%)
Fever	269 (43.5)
Cough without phlegm	201 (32.5)
Cough with phlegm	433 (70.0)
Sore throat	357 (57.7)
Runny nose	384 (62.0)
Difficulty in breathing	44 (7.1)
Chest pain during inspiration	43 (6.9)
Hoarseness of voice	244 (39.4)
Blocked nose	258 (41.7)
No symptoms	1 (0.2)
Urinary incontinence during coughing	152 (24.6)
Persistent cough that affects reading of the Quran	213 (34.4)
Persistent cough that affects prayers	285 (46.0)
Lack of energy owing to severe infection	133 (21.5)
Loss of appetite	134 (21.6)
Challenging to participate in spiritual practices	203 (32.8)
No implications	122 (19.7)
Total	619 (100)

### *Bivariate analysis of factors associated with self-reported respiratory symptoms*

The bivariate analysis of factors associated with self-reported respiratory symptoms during Hajj is presented in Table 4. Respondents who reported respiratory symptoms were slightly older than those who did not report symptoms, with a mean age of 51.7 years compared with 49.6 years among asymptomatic respondents (*P* = 0.034). There was no significant association between gender and self-reported respiratory symptoms (*P* = 0.509).

**Table 4:** Factors associated with self-reported respiratory symptoms among Malaysian Hajj pilgrims in 2023.

Variable	No, n (%)	Yes, n (%)	Total	*P*-value
Gender				
• Male	42 (18.0)	191 (82.0)	233 (31.4)	0.509
• Female	82 (16.1)	428 (83.9)	510 (68.6)	
Age (years)^a^	49.6 (12.1)	51.7 (9.4)	51.3 (9.9)	0.034^b^
Smoking status				
Nonsmoker	109 (16.1)	568 (83.9)	677 (91.1)	0.145
Smoker	8 (18.2)	36 (81.8)	44 (5.9)	
Ex-smoker	7 (31.8)	15 (68.2)	22 (3)	
Education level				
Primary	2 (50.0)	2 (50.0)	4 (0.5)	0.078
Secondary	27 (22.5)	93 (77.5)	120 (16.2)	
Diploma	16 (13.4)	103 (86.6)	119 (16)	
Degree	47 (14.5)	277 (85.5)	324 (43.6)	
Postgraduate	32 (18.2)	144 (81.8)	176 (23.7)	
Income level				
Low (B40)	34 (21.7)	123 (78.3)	157 (21.1)	0.087
Middle (M40)	39 (13.5)	249 (86.5)	288 (38.8)	
High (T20)	51 (17.1)	247 (82.9)	298 (40.1)	
BMI^a^	26.4 (5.0)	26.7 (4.8)	26.6 (5.2)	0.571
Chronic disease				
No	102 (19.4)	424 (80.6)	526 (70.8)	0.002^b^
Yes	22 (10.1)	195 (89.9)	217 (29.2)	
Diabetes mellitus				
No	115 (17.7)	535 (82.3)	650 (87.5)	0.053
Yes	9 (9.7)	84 (90.3)	93 (12.5)	
High cholesterol level				
No	111 (17.9)	510 (82.1)	621 (83.6)	0.051
Yes	13 (10.7)	109 (89.3)	122 (16.4)	
Heart disease				
No	124 (17.1)	603 (82.9)	727 (97.8)	0.070
Yes	0 (0)	16 (100)	16 (2.2)	
Chronic renal disease				
No	123 (16.6)	617 (83.4)	740 (99.6)	0.439
Yes	1 (33.3)	2 (66.7)	3 (0.4)	
Chronic lung disease				
No	124 (16.7)	619 (83.3)	743 (100)	-
Cancer				
No	123 (16.7)	615 (83.3)	738 (99.3)	0.842
Yes	1 (20)	4 (80)	5 (0.7)	
Performed Hajj/Umrah previously				
No	21 (14.1)	128 (85.9)	149 (20.1)	0.342
Yes	103 (17.3)	491 (82.7)	594 (79.9)	
Vaccinated other than meningococcal				
None	13 (23.2)	43 (76.8)	56 (7.5)	0.022^b^
Influenza	9 (18)	41 (82)	50 (6.7)	
Pneumococcal	17 (28.8)	42 (71.2)	59 (7.9)	
Influenza and pneumococcal	85 (14.7)	493 (85.3)	578 (77.8)	
Supplement use				
No	42 (23.7)	135 (76.3)	177 (23.8)	0.004^b^
Yes	82 (14.5)	484 (85.5)	566 (76.2)	
Hand hygiene practice (wash hands with water and soap)				
No	57 (21.5)	208 (78.5)	265 (35.7)	0.009^b^
Yes	67 (14)	411 (86)	478 (64.3)	
Frequent hand sanitizer use				
No	86 (15.6)	467 (84.4)	553 (74.4)	0.156
Yes	38 (20)	152 (80)	190 (25.6)	
Face mask use in public places				
No	39 (25.7)	113 (74.3)	152 (20.5)	0.001^b^
Yes	85 (14.4)	506 (85.6)	591 (79.5)	
Rinse mouth with water				
No	107 (16.9)	528 (83.1)	635 (85.5)	0.775
Yes	17 (15.7)	91 (84.3)	108 (14.5)	
Rinse mouth with antiseptic mouth rinses				
No	91 (15.9)	482 (84.1)	573 (77.1)	0.278
Yes	33 (19.4)	137 (80.6)	170 (22.9)	
Eat a balanced diet				
No	81 (17.6)	378 (82.4)	459 (61.8)	0.373
Yes	43 (15.1)	241 (84.9)	284 (38.2)	
Preventive measures				
Take measures	111 (15.7)	598 (84.3)	709 (95.4)	0.001^b^
Not taking any measures	13 (38.2)	21 (61.8)	34 (4.6)	

Abbreviation: BMI, body mass index.

a Mean (SD).

b Significant association, *P* < 0.05.

Among comorbidities, having at least one chronic disease was significantly associated with self-reported respiratory symptoms. Symptoms were reported by 195 of 217 respondents (89.9%) with chronic disease compared with 424 of 526 respondents (80.6%) without chronic disease (*P* = 0.002). Diabetes mellitus and hypercholesterolemia showed borderline associations but did not reach statistical significance at the *P* < 0.05 level. Respiratory symptoms were reported by 84 of 93 respondents (90.3%), with diabetes mellitus compared with 535 of 650 (82.3%) without diabetes mellitus (*P* = 0.053), and by 109 of 122 respondents (89.3%), with hypercholesterolemia compared with 510 of 621 (82.1%) without hypercholesterolemia (*P* = 0.051).

Several preventive practices were also associated with self-reported respiratory symptoms in bivariate analysis. Respondents who reported receiving influenza and/or pneumococcal vaccination had different proportions of respiratory symptoms compared with those who did not receive these vaccines (*P* = 0.022). Self-reported respiratory symptoms were also more commonly reported among those who took supplements (85.5% vs 76.3%; *P* = 0.004), washed hands with water and soap (86.0% vs 78.5%; *P* = 0.009), and wore face masks in public places (85.6% vs 74.3%; *P* = 0.001). However, these associations should be interpreted cautiously because the cross-sectional design cannot determine whether these practices were adopted before or after symptom onset. Using hand sanitizer, rinsing the mouth with water, rinsing with antiseptic mouth rinse, and eating a balanced diet were not significantly associated with self-reported respiratory symptoms in bivariate analysis

### *Multivariable analysis of factors associated with self-reported respiratory symptoms*

In multiple logistic regression analysis shown in Table 5, having at least one chronic disease remained independently associated with higher odds of self-reported respiratory symptoms. Respondents with chronic disease had 2.3 times higher odds of reporting respiratory symptoms than those without chronic disease (AOR 2.3; 95% CI 1.4-3.7; *P* = 0.001). Supplement use was also associated with higher odds of reporting respiratory symptoms (AOR 1.7; 95% CI 1.1-2.6; *P* = 0.015). Similarly, respondents who reported washing their hands with water and soap had higher odds of reporting respiratory symptoms (AOR 1.5; 95% CI 1.0-2.3; *P* = 0.045), although the lower CI was close to 1.0, indicating that this association should be interpreted cautiously. Regular use of hand sanitizer was associated with lower odds of self-reported respiratory symptoms (AOR 0.6; 95% CI 0.4-0.9; *P* = 0.020). This suggests that respondents who used hand sanitizer frequently had approximately 40% lower odds of reporting respiratory symptoms than those who did not. Wearing a face mask in public places was associated with higher odds of self-reported respiratory symptoms (AOR 2.2; 95% CI 1.4-3.5; *P* = 0.001).

**Table 5:** Multivariable logistic regression analysis of factors associated with self-reported respiratory symptoms.

Factors	AOR (95% CI)	SE	Z statistic	*P*-value
Presence of chronic disease	2.3 (1.4-3.7)	0.6	3.2	0.001
Supplement use	1.7 (1.1-2.6)	0.4	2.4	0.015
Handwashing with water and soap	1.5 (1-2.3)	0.3	2.0	0.045
Frequent hand sanitizer use	0.6 (0.4-0.9)	0.1	-2.3	0.020
Face mask use in public places	2.2 (1.4-3.5)	0.5	3.3	0.001

Abbreviations: AOR, adjusted odds ratio; CI, confidence interval; SE, standard error.

Outcome variable: presence of at least one self-reported respiratory symptom during Hajj. Variables were adjusted for other factors included in the final multivariable logistic regression model.

## Discussion

This study provides post-pandemic Malaysian data on self-reported respiratory symptoms, preventive practices, antibiotic use, and functional impact among Hajj pilgrims. More than four-fifths of respondents reported at least one respiratory symptom during Hajj. Although these symptoms were not clinically or microbiologically confirmed, the findings are consistent with previous Hajj studies showing that respiratory symptoms remain common during mass pilgrimage. Before the COVID-19 pandemic, respiratory symptom rates among Hajj pilgrims were reported to be high, including 70.2% among South African pilgrims, 80% among French pilgrims, and 93.4% among Malaysian pilgrims [3,4]. In contrast, during the restricted 2021 Hajj season, only 2.3% of domestic pilgrims from Saudi Arabia reported respiratory symptoms [5]. That year, Hajj was limited to approximately 60,000 COVID-19-vaccinated Saudi citizens and residents, which likely reduced crowd density, international mixing, and transmission risk. Therefore, the higher proportion of respiratory symptoms observed in the present study may reflect the return of large-scale international Hajj participation in 2023, with pilgrims from diverse geographical backgrounds gathering in crowded settings. However, direct comparison with previous studies should be made cautiously because of differences in study design, symptom definitions, sampling methods, timing of data collection, and availability of laboratory confirmation. The 2023 season is particularly relevant because it represents an early post-pandemic period after the resumption of large-scale international Hajj, when preventive behaviors shaped by the COVID-19 pandemic, such as mask use, hand hygiene, and vaccination awareness, may still have influenced pilgrims’ practices.

Commonly reported respiratory symptoms among pilgrims in this study included cough with phlegm (70%), runny nose (62%), sore throat (57.7%), and fever. A previous study among Malaysian pilgrims found higher rates of these symptoms: cough (91.5%), runny nose (79.3%), fever (59.2%), and sore throat (57.1%) [6]. In contrast, a study of Arab-Israeli and Palestinian Hajj pilgrims reported lower rates of cough (53.8%) and fever (11.5%) [7]. In other words, ‘Hajj cough’ continues to be one of the most prevalent respiratory complaints reported by pilgrims globally. For a significant portion of pilgrims (32.8%), cough posed challenges to their participation in spiritual practices, with cough-induced urinary incontinence and the need to renew ablution contributing to decreased mosque attendance. This highlights the broader implications of respiratory infections beyond physical health, affecting pilgrims’ ability to engage fully in their religious duties. Persistent cough affected prayer and Quran recitation for a substantial proportion of symptomatic respondents, and nearly one-third reported difficulty participating in spiritual practices. For Hajj pilgrims, symptoms such as cough, fatigue, sore throat, and urinary incontinence during coughing may affect comfort, concentration, ablution, mosque attendance, and participation in rituals. Therefore, respiratory health during Hajj should be understood not only as infection prevention but also as part of supporting pilgrims’ ability to fulfill religious obligations with dignity and wellbeing.

Chronic disease was independently associated with higher odds of self-reported respiratory symptoms. This finding is clinically plausible because many Hajj pilgrims are older adults and may have underlying comorbidities that increase their susceptibility to illness, symptom severity, healthcare seeking, and complications during physically demanding pilgrimage activities [8]. In bivariate analysis, diabetes mellitus and hypercholesterolemia showed borderline associations with respiratory symptoms. The association between chronic disease and respiratory symptoms reinforces the importance of pre-Hajj health optimization. Prospective pilgrims should be supported to achieve good control of chronic illnesses, maintain medication adherence, assess their fitness to travel, and seek early medical care if symptoms develop during pilgrimage. Pre-travel consultations also provide an opportunity for healthcare providers to identify higher-risk individuals and deliver targeted counseling on vaccination, respiratory hygiene, medication planning, hydration, and avoidance of excessive physical strain. This issue has become increasingly relevant in recent Hajj planning. For Hajj 2026, Saudi Arabia has introduced stricter health requirements, including the need for pilgrims to be free from serious or uncontrolled chronic diseases and be physically able to perform Hajj rituals independently. Conditions such as chronic kidney failure, serious heart disease, chronic lung or liver disease, active infection, cognitive or motor impairment, high-risk pregnancy, and cancer under treatment have been reported as among the conditions affecting eligibility [9]. These measures reflect growing recognition that pre-Hajj health screening and optimization are essential to reduce morbidity and mortality during pilgrimage.

Antibiotic use was common among respondents who reported respiratory symptoms, although 41.2% did not report receiving antibiotics. Because this study did not include clinical assessment, prescription review, or microbiological testing, the appropriateness of antibiotic use could not be determined. Respiratory symptoms during Hajj may be caused by viral, bacterial, or non-infectious conditions, and many episodes are likely to be mild and self-limiting [10]. However, bacterial infection and complications cannot be excluded based on self-reported symptoms alone. These findings highlight the need for balanced health education before and during Hajj. Pilgrims should be advised to carry appropriate symptomatic relief, such as antipyretics, cough remedies, throat lozenges, and adequate hydration strategies. At the same time, healthcare providers should continue to promote prudent antibiotic use and avoid unnecessary antibiotic prescribing for uncomplicated respiratory symptoms, while ensuring timely assessment and treatment for pilgrims with severe, persistent, or worsening symptoms

Several preventive practices showed associations with self-reported respiratory symptoms, but these findings require cautious interpretation. Frequent hand sanitizer use was associated with lower odds of reporting symptoms, supporting the continued promotion of accessible hand hygiene during Hajj. In contrast, face mask use, supplement use, and handwashing with water and soap were associated with higher odds of reporting symptoms in the adjusted model. These results should not be interpreted as evidence that these practices caused respiratory symptoms. A more plausible explanation is reverse causality or health-seeking behavior, whereby pilgrims may have adopted masks, supplements, or more frequent handwashing after developing symptoms or perceiving themselves to be at risk. The survey also did not assess timing of practice in relation to symptom onset, consistency of use, mask type, mask fit, frequency of mask replacement, and type of supplement. Previous Hajj studies have also shown mixed findings on the effectiveness of face masks. Some studies reported that face mask use was not clearly protective against laboratory-confirmed or clinical respiratory infections, whereas inconsistent use and poor adherence may reduce its effectiveness [6,11]. Conversely, regular replacement of face masks has been associated with lower respiratory symptom occurrence among Hajj pilgrims, suggesting that proper mask practice may be more important than mask use alone [12]. These findings highlight the need for future studies to collect more detailed information on the timing, consistency, and quality of mask use. Overall, the findings support a balanced approach to respiratory infection prevention during Hajj. Pilgrims should be educated not only to adopt preventive measures but also to practice them correctly and consistently. Health education should emphasize proper hand hygiene, correct mask use and replacement, cough etiquette, avoidance of crowded poorly ventilated spaces where possible, and early care seeking for severe or persistent symptoms. Gargling or mouth rinsing practices were uncommon among respondents, and their potential role in respiratory hygiene may warrant further evaluation in future Hajj health education programs [13].

Vaccination uptake beyond the mandatory meningococcal vaccine was high in this study, with most respondents reporting receipt of influenza and/or pneumococcal vaccines. This contrasts with earlier pre-pandemic findings among Malaysian Hajj and Umrah pilgrims, where uptake of influenza and pneumococcal vaccines was much lower at 28.6% and 25.4%, respectively [14]. The higher uptake observed in the present study may reflect increased health awareness after the COVID-19 pandemic, stronger pre-travel health messaging, and greater acceptance of respiratory infection prevention measures. However, these findings should be interpreted cautiously. Vaccination status was self-reported, and this study was not designed to assess vaccine effectiveness. Therefore, no causal conclusion can be made regarding the protective effect of vaccination against respiratory symptoms, severity, or hospitalization. Future studies should verify vaccination records and include clinical outcomes such as healthcare visits, hospitalization, and laboratory-confirmed respiratory infections.

This study has several limitations. First, the use of convenience sampling through WhatsApp groups may have introduced selection bias and limits generalizability to all Malaysian Hajj pilgrims in 2023. Pilgrims without access to smartphones, WhatsApp, or online survey platforms may have been under-represented. Second, respiratory symptoms were self-reported retrospectively and were not clinically or microbiologically confirmed; therefore, the findings should not be interpreted as confirmed RTI, influenza, COVID-19, or bacterial infection. Third, the cross-sectional post-Hajj design prevents assessment of temporality, particularly for preventive practices that may have been adopted after symptom onset. Fourth, recall bias is possible because respondents were asked to report symptoms, practices, and antibiotic use after the pilgrimage. Despite these limitations, the study provides useful post-pandemic reference data on respiratory symptoms, preventive practices, antibiotic use, and worship-related impact among Malaysian Hajj pilgrims.

In conclusion, self-reported respiratory symptoms were common among surveyed Malaysian Hajj pilgrims in 2023. Chronic disease was independently associated with higher odds of reporting symptoms, whereas frequent hand sanitizer use was associated with lower odds. Antibiotic use was common among symptomatic respondents, although the appropriateness of prescribing could not be assessed. Because this study used an online convenience sample and retrospective self-report, the findings should not be interpreted as the true national prevalence of RTI among all Malaysian pilgrims. Nevertheless, the findings support strengthening pre-Hajj health optimization, respiratory hygiene education, targeted prevention for pilgrims with chronic disease, symptomatic relief planning, and prudent antibiotic use during Hajj.

## CRediT authorship contribution statement

**Nurul Azmawati Mohamed:** Writing – review & editing, Supervision, Project administration, Conceptualization. **Shalinawati Ramli:** Writing – original draft, Data curation. **Nuurain Amirah Mohd Razi:** Methodology, Formal analysis.

## Declaration of competing interest

The authors have no competing interests to declare.
